# Orthodontic management of a non-syndromic patient with concomitant bimaxillary hypohyperdontia: a case report

**DOI:** 10.1590/2177-6709.25.1.036-046.oar

**Published:** 2020

**Authors:** Ei Ei Hsu Hlaing, Yoshihito Ishihara, Atsuro Fujisawa, Takashi Yamashiro, Hiroshi Kamioka

**Affiliations:** 1 Okayama University, Graduate School of Medicine, Dentistry and Pharmaceutical Sciences, Department of Orthodontics (Okayama, Japan).; 2 Okayama University Hospital, Department of Orthodontics (Okayama, Japan).; 3 Osaka University, Graduate School of Dentistry, Department of Orthodontics and Dentofacial Orthopedics (Suita, Japan).

**Keywords:** Agenesis, Hypodontia, Supernumerary teeth

## Abstract

**Introduction:**

Tooth agenesis is one of the most common dental anomalies; however, the concomitant occurrence of opposite dental numerical variation of hypohyperdontia is extremely rare.

**Objective:**

To report the successful orthodontic management of a patient with non-syndromic concomitant bilateral agenesis of mandibular canines and two midline inverted supernumerary maxillary teeth.

**Case report:**

21-year-old female patient with a chief complaint of protrusive right maxillary central incisor. The patient was diagnosed with a mild Class II skeletal base, Angle Class III molar relationship and increased overjet associated with hypohyperdontia. Anterior open bite accompanied with tongue-thrusting habit were also observed. Two temporary anchorage devices (TADs) were implanted at the buccal side of the maxillary molar region to control vertical height. Anterior teeth retraction was done after extraction of the maxillary first premolars, to improve the excessive overjet. The treatment mechanics involved lingual brackets system for the maxillary arch and transpalatal arch for anchorage control.

**Results:**

The total active treatment period was 35 months. Acceptable occlusion with increased bite force and contact area as well as functional excursion were established without interference, following complex orthodontic treatment with premolar substitution. The resultant occlusion and a satisfactory facial profile were maintained after 29 months of retention.

**Conclusion:**

The present case report provides implications regarding the orthodontic treatment of hypohyperdontia-associated substitution for missing teeth as an effective option for improving aesthetic and functional aspects.

## RESUMO

**Introdução:**

A agenesia é uma das anomalias dentárias mais comuns; porém, a ocorrência concomitante de variações numéricas do tipo hipo-hiperdontia em ambas as arcadas é extremamente rara.

**Objetivo:**

Descrever o tratamento ortodôntico bem-sucedido de uma paciente não sindrômica que apresentava agenesia bilateral dos caninos inferiores e dois dentes supranumerários na região de linha média superior.

**Descrição:**

Paciente do sexo feminino com 21 anos de idade, apresentando queixa principal de protrusão dos incisivos centrais superiores. Foi diagnosticada com leve Classe II esquelética, relação de molares em Classe III de Angle, sobressaliência aumentada e hipo-hiperdontia. Além disso, a paciente apresentava mordida aberta anterior associada ao hábito de interposição lingual. Dois dispositivos de ancoragem temporária (DATs) foram instalados por vestibular na região dos molares superiores, para controle vertical. A retração dos dentes anteriores foi feita após a extração dos primeiros pré-molares superiores, para reduzir a sobressaliência acentuada. A mecânica adotada envolveu o uso de braquetes linguais na arcada superior e barra transpalatina para controle da ancoragem.

**Resultados:**

Após 35 meses de fase ativa desse tratamento complexo, com substituição dos pré-molares, obteve-se oclusão aceitável, com aumento da força oclusal e da área de contatos oclusais, bem como movimentos excursivos funcionais bem estabelecidos e sem interferências. Após 29 meses de contenção, verificou-se estabilidade da oclusão e do perfil facial.

**Conclusão:**

Esse relato de caso clínico mostra que o tratamento ortodôntico com substituição de dentes ausentes associados à hipo-hiperdontia é uma opção efetiva para a melhora dos aspectos estéticos e funcionais.

## INTRODUCTION

Tooth agenesis is one of the most common dental anomalies in permanent dentition. The number of teeth present is assessed when making an orthodontic diagnosis because congenitally missing teeth might often be challenging to manage in clinical orthodontics. The prevalence of dental agenesis in permanent dentition, excluding the third molars, is about 4.5% to 7.4% in Caucasians, and 0.2% to 16.2% in Asians.^1^ Past studies have reported that the prevalence rate is higher in females than in males.^2^

The patterns of associated tooth agenesis can be classified, according to the number of missing teeth, as anodontia (complete absence of teeth), oligodontia (six or more missing teeth) and hypodontia (fewer than six missing teeth), and its prevalence is strongly influenced by ethnicity.^3^ Agenesis can occur as non-syndromic or associated with genetic syndromes. Hypodontia and supernumerary teeth simultaneously found in the same individual is referred to as concomitant hypohyperdontia or oligopleiodontia.^4^

Lateral incisors and premolars are the most common congenitally missing teeth, and premaxilla is the most frequently involved area with supernumerary teeth in concomitant hypohyperdontia.^5^ On the other hand, bilateral agenesis of permanent mandibular canines is extremely rare^6^ and is a particularly challenging situation for clinicians, due to the special considerations required to achieve an optimum aesthetic and functional result, including space management, discrepancy of the intermaxillary tooth materials, and stable occlusion. Treatment options for managing congenitally missing mandibular canines may either include opening the space for prosthesis restoration or closing the space to establish occlusion, with premolar substitution. However, few reports have described the clinical details concerning the orthodontic approach in such patients.^7^ ^,^ ^8^

The present case report describes the successful orthodontic treatment of a patient with non-syndromic concomitant bimaxillary hypohyperdontia (bilateral agenesis of mandibular canines and two midline inverted supernumerary maxillary teeth) and increased overjet and anterior open bite. A satisfactory treatment outcome was obtained using a lingual brackets system for the upper arch in combination with a skeletal anchorage system and transpalatal arch for vertical and anchorage control.

## CASE REPORT

**Figure 1:**
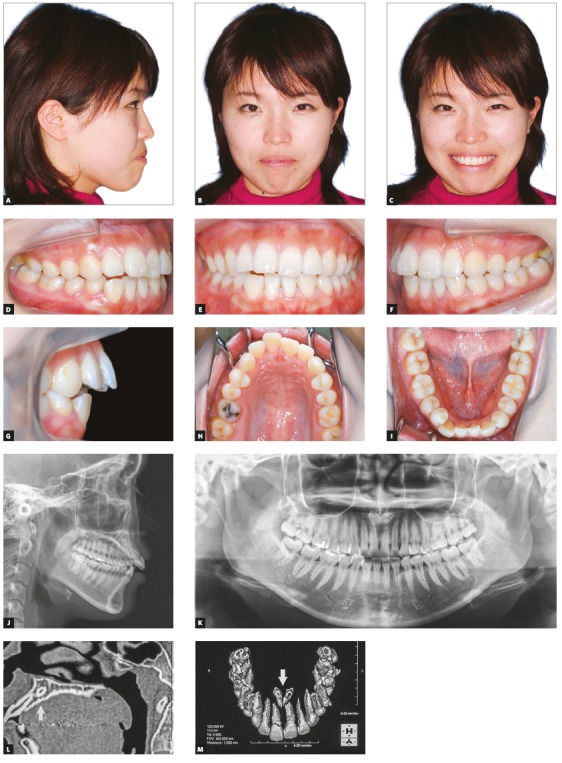
Pretreatment records (at 21 years and 3 months of age): A-I) extraoral and intraoral photographs; J) lateral cephalometric radiograph; K) panoramic radiograph; L-M) cone-bean computed tomography to assess the supernumerary teeth in the maxillary arch.

A 21-year-old woman visited the outpatient orthodontic clinic of Okayama University Hospital with the chief complaint of a protruding maxillary right central incisor. She desired nonsurgical treatment with invisible appliance. Front and profile facial photographs showed a symmetrical face, convex profile and protruded and incompetent lips (Fig 1). Mentalis muscle strain was seen, and a slightly gummy smile was observed. An intraoral examination revealed an excessive 8.0-mm overjet (OJ), with Angle Class III molar relationships on both sides and bilateral congenitally missing mandibular canines. A Class II division 1 incisor relationship, with anterior open bite (AOB) and tongue thrusting habit was observed. She had no family history of the same open bite condition. Anterior mild crowding was also observed in maxillary and mandibular arches. The upper dental midline almost coincided with the facial midline; however, the lower dental midline was shifted 1.0 mm toward left. Panoramic radiograph confirmed the absence of mandibular canines on both sides, and two midline supernumerary teeth were detected at the maxillary central incisors region (Fig 1K). Cone-beam computed tomography showed no pathological problems in the root structure of maxillary central incisors on either side (Figs 1L to 1M). The patient reported clicking sounds in the temporomandibular joint without pain during maximum opening on the right side. The interincisal distance on maximum opening was 44 mm.

In comparison to the Japanese female norms^9^, cephalometric evaluation showed a mild skeletal Class II jaw relationship (ANB = 4.5°; SNA = 83.0°; SNB = 78.5°). Although her lower facial height ratio was normal (N-Me = 125.5 mm; Me-PP = 71.5 mm), with normal mandibular plane angle (FMA = 29.0°) (Fig. 1J, Table 1), a decreased overbite of -1.0 mm was observed, since she had two distinct upper occlusal planes. The maxillary incisor angle was increased (U1-SN = 114.0°), and the mandibular incisor angle was slightly reduced (L1-Mp = 87.5°). The lower lip was slightly protruded against the aesthetic E-line (upper = 0.0 mm; lower = +2.5 mm). Given the above findings, the patient was diagnosed with a mild skeletal Class II jaw-base relationship; increased OJ, due to the bilateral congenitally missing mandibular canines; and Angle Class III malocclusion with AOB.

**Table 1:** Summary of cephalometric measurements at the three treatment stages.

Variable	Japanese norms for women	SD	Pretreatment	Posttreatment	2.5 years post-retention
Angular (degrees)					
ANB	2.8	2.44	4.5	3.5	3.5
SNA	80.8	3.61	83.0	81.5	81.5
SNB	77.9	4.54	78.5	78.0	78.0
FMA	30.5	3.6	29.0	29.0	29.5
Go.A	122.1	5.29	122.5	121.5	121.5
U1-SN	105.9	8.79	114.0	97.0	97.0
L1-Mp	93.4	6.77	87.5	94.0	94.0
Interincisal Angle	123.6	10.64	123.0	132.0	132.0
Linear (mm)					
S-N	67.9	3.65	71.0	71.0	71.0
N-Me	125.8	5.04	125.5	125.5	127.0
Me-PP	68.6	3.71	71.5	71.5	72.5
Ar-Go	47.3	3.33	49.0	49.0	49.5
Go-Me	71.4	4.14	75.0	75.0	75.0
Ar-Me	106.6	5.74	111.5	111.5	112.0
Overjet	3.1	1.07	8.0	2.0	2.0
Overbite	3.3	1.89	-1.0	2.0	1.5
U1/PP	31	2.34	31.0	31.0	31.0
U6/PP	24.6	2	26.0	25.0	25.5
L1/Mp	44.2	2.68	44.5	46.0	46.0
L6/Mp	32.9	2.5	36.5	36.5	38.0

### Treatment objectives and alternatives

The primary treatment objectives were to correct the increased OJ with substitution of the mandibular canines by the first premolars, and the AOB without extruding the maxillary and mandibular incisors; create lip sealing and improve facial aesthetics. Additional objectives included Class III molar correction and establishment of functional occlusion. Based on the primary objectives, extraction of the maxillary first premolars was considered to correct the OJ. The use of temporary anchorage devices (TADs) was also planned to control the vertical relationship of maxillary molars.

Another conservative approach involves maxillary molar distalization using TADs, and extraction of the maxillary third molars. However, this option increases the risk of AOB, since vertical control is difficult. After a thorough discussion of these options, the patient agreed with the premolar extraction treatment plan, with lingual brackets in the maxillary arch and labial brackets in the mandibular arch.

### Treatment progress

**Figure 2:**
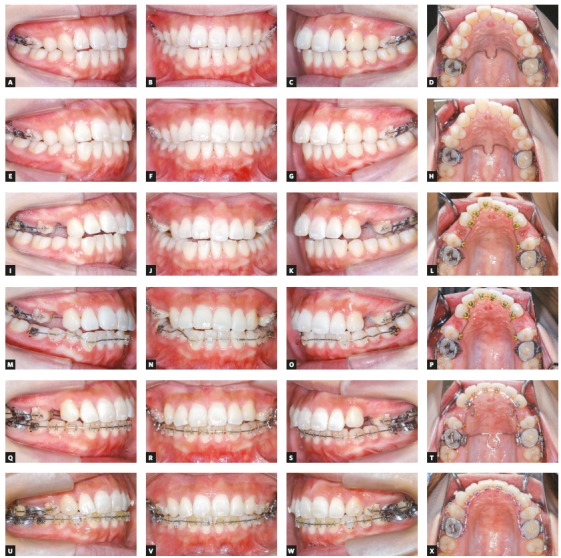
Series of intraoral photographs taken during treatment: A-D) start of treatment; E-H) molar intrusion; I-L) leveling of the maxillary arch; M-P) leveling of the mandibular arch; Q-T) space closure of the maxillary arch; U-X) detailing.

After extraction of two supernumerary teeth, a transpalatal arch was inserted in the maxillary arch (Figs 2A - 2D). Two TADs were also implanted at the buccal side of the maxillary molar region of both sides, to control the vertical relationship of posterior teeth. Four weeks after implantation, a 0.018-in preadjusted Edgewise appliance (iPass^®^, Dentaurum, Pforzheim, Germany) was placed, with sectional 0.016 × 0.022-in NiTi wires in the maxillary posterior segment. Two months after leveling and alignment, 0.016 × 0.022-in stainless steel (SS) archwire was installed to initiate the vertical anchorage control of the maxillary molars, using an elastomeric chain from the TADs to the segmental wires (Figs 2E - 2H). After 4 months of molar intrusion and bilateral maxillary first premolars extraction, the maxillary lingual brackets (STb^®^, Ormco, Glendora, CA, USA) by indirect bonding system and the mandibular labial brackets (iPass^®^, Dentaurum, Pforzheim, Germany) by direct bonding system were placed, then the alignment was initiated by inserting 0.014-in NiTi wires in both arches (Figs 2I - 2P).The wire size was increased to 0.016 × 0.022-in NiTi. For space closure and anterior teeth retraction of the maxillary arch, a loop mechanic with 0.017 × 0.025-in SS was used (Figs 2Q - 2T). Detailing and finishing were achieved using 0.016 × 0.022-in β-titanium-molybdenum wire in both arches (Figs 2U - 2X). After 35 months of active treatment, a circumferential double retainer with a tongue crib in the maxillary arch and a lingual bonded retainer in the maxillary and mandibular arches were used as retention appliances. 

### Treatment results

**Figure 3:**
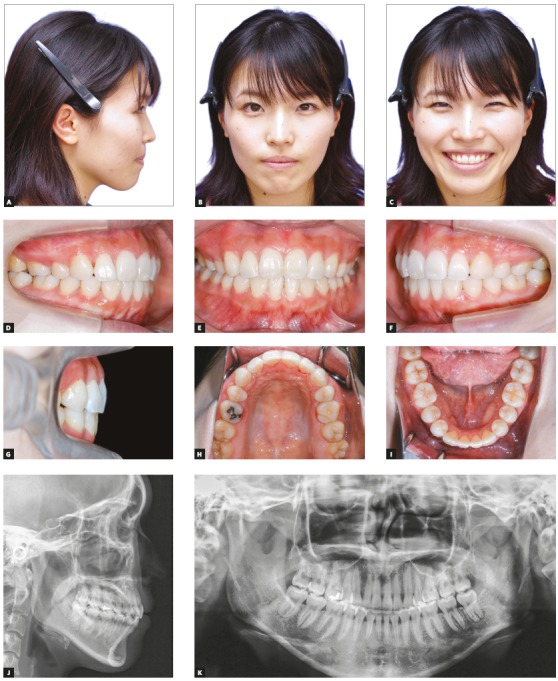
Posttreatment records (at 24 years and 2 months of age): A-I) extraoral and intraoral photographs; J) lateral cephalometric radiograph; K) panoramic radiograph.

**Figure 4:**
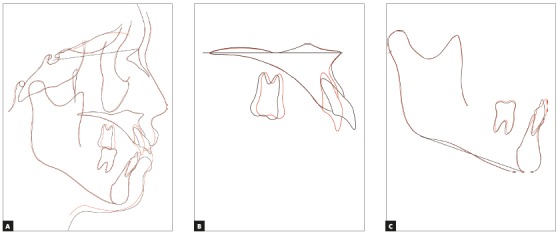
Superimposed pretreatment (black line) and posttreatment (red line) cephalometric tracings: A) superimposed on the sella-nasion plane at the sella; B) superimposed on the palatal plane at the ANS; C) superimposed on the mandibular plane at the menton.

Compared to the pretreatment photographs, the posttreatment photographs showed retraction of the upper and lower lips, which subsequently improved the patient’s facial profile and the relief of lip incompetence (Figs 3A-3I). Although the missing mandibular canines were substituted by mandibular first premolars, well-aligned arches and good interdigitation were achieved, with Angle Class I molar relation (Figs 3A-3I). By means of the extraction of maxillary first premolars and retraction of maxillary anterior teeth, an ideal overjet of 2.0 mm and an overbite of 2.0 mm were also achieved (Table 1). The posttreatment panoramic radiograph showed acceptable root parallelism, with no significant root resorption, except for slight apical root resorption of the maxillary right second premolar (Fig 3K). Superimposition of the pre- and posttreatment cephalometric tracings showed that the maxillary and mandibular incisors inclination was normalized (Fig 4). The mild skeletal Class II relationship was improved due to the slight reduction in the SNA angle (Table 1). The vertical dimension was maintained after orthodontic treatment. 

**Figure 5:**
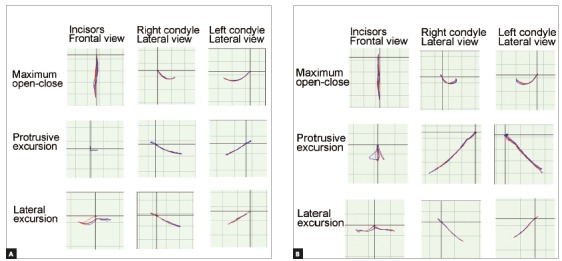
Condylar movement and incisal paths recorded using a jaw movement recording system with six degrees of freedom. The red lines indicate the opening phase, and the blue lines indicate the closing phase: A) pretreatment, B) posttreatment.

**Figure 6:**
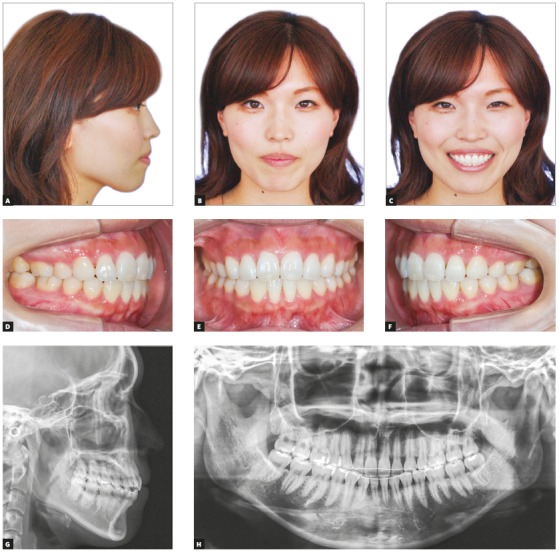
Post-retention records (at 26 years and 7 months of age): A-F) extraoral and intraoral photographs, G) lateral cephalometric radiograph, H) panoramic radiograph.

**Figure 7:**
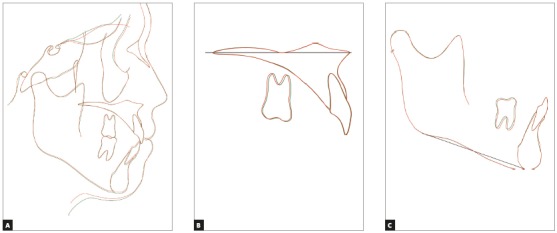
Superimposed posttreatment (red line) and post-retention (green line) cephalometric tracings: A) superimposed on the sella-nasion plane at the sella; B) superimposed on the palatal plane at the ANS; C) superimposed on the mandibular plane at the menton.

In the evaluation using a jaw movement recording system with six degrees of freedom (Gnathohexagraph System, version 1.31; Ono Sokki, Kanagawa, Japan)^10^, a smooth increase in the condylar movement was observed during protrusive or lateral excursion (Fig 5, Table 2).The interincisal distance on maximum opening without pain was maintained at 45 mm. Good facial aesthetics and acceptable occlusion were maintained after 29 months of retention (Figs 6 and 7). Occlusal force and occlusal contact area were also increased, compared with before treatment (Table 3). The patient was satisfied with treatment results.

**Table 2:** The condylar and lower incisor path lengths (mm) during mandibular movements.

	Maximum open-close			Protrusive excursion			Lateral excursion		
	Lower incisor	Right condyle	Left condyle	Lower incisor	Right condyle	Left condyle	Lower incisor	Right condyle	Left condyle
Pretreatment	40.92	13.83	20.43	8.04	2.46	3.7	16.73	10.1	8.82
Posttreatment	45.03	10.42	15.59	9.38	8.71	9.38	20.1	11.78	11.23

## DISCUSSION

Congenital agenesis is a life-long problem, and agenesis of the canines in the permanent dentition is very rare.^11^ It affects the maxillary arch more often than the mandibular arch, with a reported incidence of 0.1% in the maxilla and 0.02% in the mandible.^12^ Some studies have suggested genetic or familial inheritance as a significant etiological factor of tooth agenesis.^13^ Familial inheritance is transmitted as an autosomal dominant, recessive or X-linked recessive pattern of inheritance.^14^ Some regulatory homeobox genes, such as Muscle Segment Box (MSX) 1, MSX 2, PAX 9 and TGFA, play a role in dental development and craniofacial morphogenesis.^15^ Mutations in the homeobox of human MSX 1 are responsible for a specific pattern of inherited tooth agenesis.^16^

Tooth agenesis can be associated with other dental anomalies, such as supernumerary teeth or cleft lip and palate.^17^ The concomitant occurrence of hypohyperdontia is between 0.002% and 3.1% and is rare in the non-syndromic population.^18^ This numerical dental anomaly is reportedly more common in permanent dentition than in primary dentition.^19^ Despite evidence that it may be derived from interactions between the epithelial and mesenchymal cells in the initiation stage of odontogenesis or disturbances in the migration, proliferation and differentiation of neural crest cells, the exact etiology remains unknown.^17^ Hypohyperdontia can be divided into mandibular, maxillary with premaxillary subtype, and bimaxillary hypohyperdontia.^20^

There are several clinical presentations of concomitant hypohyperdontia. However, while a wide range of treatment approaches have been proposed,^21^ little information is available regarding the clinical and functional assessment of these factors in orthodontic patients. Orthodontic treatment for patients with hypohyperdontia is quite complex and requires thorough planning based on the patient’s existing malocclusion, as it may result in changes to the timing and/or sequence of tooth eruption, including delayed exfoliation or retention of deciduous teeth, transposition, ectopic eruption of other teeth, root resorption of adjacent teeth and space problems for permanent dentition.^22^

The present case displayed hypodontia (bilateral agenesis of permanent mandibular canines) and hyperdontia (two inverted supernumerary teeth between the maxillary central incisors) with increased OJ and AOB. Orthodontic space closure can produce better results, with stable occlusion and promote periodontal health, than prosthetic replacement.^23^ However, some cases result in increased OJ and overbite, due to Bolton’s tooth size discrepancy.^24^

The present patient wished to wear lingual brackets in the maxillary arch, for aesthetic reasons. While lingual brackets treatment has some limitations with regard to torque control of anterior teeth^25^ and vertical control of posterior teeth,^26^ the treatment outcome of AOB is more stable than with labial brackets treatment or surgery.^27^ For this reason, the present patient was treated with lingual brackets in the maxillary arch and labial brackets in the mandibular arch. 

Another concern for orthodontists is the stability of open bite treatment, due to the difficulty in resolving vertical discrepancy, eliminating the tongue thrust habit and controlling tongue posture.^28^ We used an approach for open bite correction by intruding maxillary molars with miniscrew-aided mechanics prior to correcting anteroposterior discrepancy. Such separate step-by-step treatment procedures of the anterior and posterior segments appeared to be one of the reasons for the prolonged overall treatment duration (35 months). The present treatment outcome was relatively stable, with harmonious occlusal relationship during the 29-month post-retention period. Regarding the posttreatment cephalometric analysis, the vertical dimension was maintained throughout the treatment. 

Assessments of the function of substituted teeth revealed that the patient’s condylar movement and incisal pathway of protrusive, lateral excursion had improved after orthodontic treatment. A smooth pathway of protrusive and lateral excursion is crucial for achieving functionally stable occlusion.^29^ As an additional outcome, a gnathohexagraphic analysis showed that her chewing function was improved with the increased occlusal force and contact area (Table 3). These observed functional improvements might be attributed to the correction of dental numerical anomalies, resulting in functional occlusion and more stable jaw movement. However, a long-term follow-up and a longitudinal assessment are required to confirm the efficacy of substituting a canine by a premolar, and the effects of this treatment on periodontal health. Further studies are also needed to assess the functional overload on the substituted mandibular first premolars. 

**Table 3:** Changes in the occlusal force and occlusal contact area during the orthodontic treatment.

	Occlusal force (N)	Occlusal contact area (mm^2^)
Pretreatment	624	15.1
Posttreatment	528	10.5
Postretention	734	16.2

Although the present case report describes the congenitally missing mandibular canines, there is controversy as regards to the missing teeth. Recent studies reported that the second most frequently missing teeth in hypodontia cases was the lateral incisor, excluding third molars.^5,30^ Thus, the missing teeth in the present case report could be the mandibular lateral incisors, since there was morphological variation and slight incisal wear of the present teeth. We think that this does not seems to affect the treatment options for achieving an acceptable occlusion. 

## CONCLUSION

The present case described the orthodontic management involving the substitution of missing canines by first premolars, in a patient with bilateral agenesis of mandibular canines. This treatment approach with first premolar substitution is an effective option to restore the functional requirements and approximate a natural-looking intact dentition, achieving proper esthetics for treating the patients with congenitally missing mandibular canines.
