# ERRATUM

**DOI:** 10.1590/2177-6709.25.1.036-046.err

**Published:** 2020

The original article *“Orthodontic management of a non-syndromic patient with concomitant bimaxillary hypohyperdontia: a case report”*, with DOI: 10.1590/2177-6709.25.1.036-046.oar, published in Dental Press J Orthod, vol. 25, no. 1, Jan./Fev. 2020, Epub Mar 20, 2020, has the following authors:

Ei **Ei Hsu Hlaing**, Yoshihito **Ishihara**, Atsuro **Fujisawa**, 

Takashi **Yamashiro**, Hiroshi **Kamioka**

The “**How to cite this article**” presented the following information:

Hlaing Ei Ei Hsu, Ishihara Yoshihito, Fujisawa Atsuro, Yamashiro Takashi, Kamioka Hiroshi. Orthodontic management of a non-syndromic patient with concomitant bimaxillary hypohyperdontia: a case report. Dental Press J. Orthod. [Internet]. 2020 Jan [cited 2020 Apr 15] ; 25( 1 ): 36-46.

Now the article should have the following “**How to cite this article**”: 

Ei Hsu Hlaing Ei, Ishihara Yoshihito, Fujisawa Atsuro, Yamashiro Takashi, Kamioka Hiroshi. Orthodontic management of a non-syndromic patient with concomitant bimaxillary hypohyperdontia: a case report. Dental Press J. Orthod. [Internet]. 2020 Jan [cited 2020 Apr 15] ; 25( 1 ): 36-46.

